# Diagnosis of Peanut Allergy in Preschool Children: The Impact of Skin Testing With a Novel Composition of Peanuts

**DOI:** 10.3389/fped.2021.739224

**Published:** 2021-11-30

**Authors:** Mona I. Kidon, Soad Haj Yahia, Diti Machnes-Maayan, Yael Levy, Shirli Frizinsky, Ramit Maoz-Segal, Irena Offenganden, Ron S. Kenett, Nancy Agmon-Levin, Ran Hovav

**Affiliations:** ^1^Clinical Immunology, Angioedema and Allergy Unit, Center for Autoimmune Diseases, Sheba Medical Center, Tel Hashomer, Israel; ^2^Sackler School of Medicine, Tel-Aviv University, Tel Aviv, Israel; ^3^Pediatric Allergy Clinic, Safra Children's Hospital, Sheba Medical Center, Tel Hashomer, Israel; ^4^KPA Group and Institute for Drug Research, School of Pharmacy, Hebrew University, Jerusalem, Israel; ^5^Volcani Center, Plant Sciences Institute, Ministry of Agriculture, Beit Dagan, Israel

**Keywords:** peanut, allergy, diagnosis, early life, preschool, high threshold

## Abstract

Peanut allergy is an increasing concern in younger children. Available bedside diagnostic tools, i.e., prick tests with commercial extracts or peanut-containing foods have only limited predictive values. In a cohort of preschoolers with both a history of allergic reactions and sensitization to peanut proteins, we aimed to characterize the impact of skin tests with a novel composition of peanuts LPP-MH. Almost one quarter (27/110) of preschool children, with a history of allergic reactions to peanuts and positive standard IgE-mediated tests for peanut allergy, can tolerate the reintroduction of peanut proteins into their diet after resolving their allergy and, thus, can avoid adverse health outcomes associated with the false diagnosis. In the younger age group, a quarter of peanut allergic children, display a relatively high threshold, potentially enabling an easier and safer oral immunotherapy protocol in this window of opportunity in childhood. The use of the novel diagnostic skin test, LPP-MH, significantly improves the predictive value of outpatient evaluation for the outcomes of peanut challenge as well as the expected threshold at which the PA child will react, thus, making for a better informed decision of how, when, and where to challenge.

## Introduction

Peanut allergy continues to be a significant burden to children and families worldwide (1–3).

The diagnosis of peanut allergy in the clinical setting is based on a suggestive medical history upon exposure to a relevant food in conjunction with evidence of IgE-mediated sensitization via a skin prick test or peanut-specific IgE (4–6). Although 95% confidence levels for both skin tests and peanut-specific IgE have been published (7), and component resolved diagnostics (CRD) have been proposed to improve diagnostic accuracy (8–10), medically supervised oral food challenges (OFCs) are still the “gold standard” (6) for diagnosis.

When compared with challenge results, all available diagnostic tests, assessing sensitization, overestimate the occurrence of the true clinical allergy (11). Since OFCs are costly, time and resource consuming, and incurring a significant risk of severe reactions (12), they are not routinely performed in young children (13, 14), leaving the diagnosis of peanut allergy in this age group dependent upon tests with limited sensitivity, specificity, and predictive values.

In children at risk, a false-positive diagnosis incurs both severe quality of life (QOL) impairment, heightened anxiety from accidental ingestion, and may limit participation in social events, school, and work. This falsely incurred diagnosis also increases the risk of developing true life-threatening allergy secondary to -erm avoidance (15, 16). Therefore, improving the pre challenge diagnostic accuracy is a priority.

Non-standardized test with fresh food preparations, have been studied in the diagnosis of food allergy with reasonable success (17); however, prick–prick tests with peanuts (as peanut butter, for example) have not shown a vastly improved predictive value compared with the available commercial extracts (18).

Therefore, there is an acute need for the development of improved tests and better diagnostic protocols, capable of predicting the outcome of food challenges in the outpatient clinic setting.

Many peanut proteins have been identified as allergenic in different individual people and populations (19). Ara h 1, Ara h 3.01, and Ara h 3.02 (the former Ara h 4) belong to the cupin superfamily. The conglutins Ara h 2, Ara h 6, and Ara h 7, and the non-specific lipid transfer protein Ara h 9, 16, 17, and 18 belong to the prolamin superfamily. Ara h 5 (profilin) and Ara h 8 (Bet v 1-homologous protein) cause class II food allergies and are associated with inhalation allergy to pollen via the sequential and/or conformational similarity of molecules. Two peanut oleosins are listed as Ara h 10 and Ara h 11, two defensins as Ara h 12 and Ara h 13 by the WHO/IUIS Allergen Nomenclature Subcommittee, and additional relevant allergens have been and may be subsequently identified.

Therefore, the use of skin tests utilizing a whole, unprocessed peanut preparation, containing all of these potential allergens may be better suited for the accurate diagnosis of peanut allergy.

The allergenicity of peanut seeds during their progressive developmental stages was studied in a prospective, cross sectional, challenge-validated study in peanut allergic children (20). Overall, with increased maturation, there is an increase in protein content in general as well as increased production of allergenic proteins. However, at equivalent total protein concentrations, immature peanuts have significant expressions of Ara h1, Arah3, and Ara h6, as well as a relatively increased expression of the genome A copy of Arah2 (less allergenic isoform) over the B genome copy. In addition, organized protein bodies are smaller in size, with a greater dispersion throughout the cell.

The aim of our study was to explore the value of skin testing with the new lyophilized peanut preparation of immature seeds developed by the Volcani Agricultural Research Center (LPP-MH), for the diagnosis of peanut allergy in preschool children at high risk as well as estimating the minimal eliciting threshold for an allergic reaction in these children.

## Materials and Methods

### Patients

Patients were evaluated as part of a single-center, cross-sectional prospective study enrolling children with a prior history of immediate allergic reactions to peanut-containing foods and evidence of IgE sensitization to peanuts (as either positive prick tests of a positive specific IgE test to peanuts). For the purposes of our present study, preschool children 1–6 years of age, fulfilling the above criteria, were offered a series of diagnostic tests, after which all children underwent a standardized open oral food challenge (OFC). The study was conducted in the pediatric allergy clinic of the Safra Children's Hospital, Tel Hashomer, Israel, between January 2017 and July 2020.

Prior to study entry, parents and guardians were thoroughly counseled on the potential risks and benefits before they signed informed consent forms approved by the Institutional Review Board (IRB) of the Sheba Medical Center and the national IRB as required for any study involving children in Israel.

### Skin Tests

All tests were performed by the same, highly trained, pediatric allergy nurse, and recorded within the electronic medical records. Skin prick testing (SPT) was performed using single-head lancets on the forearms of children with histamine (1 mg/ml) as positive control, glycerinated saline as negative control and whole-peanut extract (ALK-Abelló, Denmark). Wheal and flare sizes were measured after 15 min. Patients abstained from anti-histamine-containing medications for at least 1 week prior to the procedure.

Prick–Prick Skin Tests were performed with LPP-MH (Volcani Agricultural Research Center, Department of Plants Genetics, Rishon Lezion, Israel).

Peanut plants were grown in a net house during summer 2017 on 1.9-m wide beds and two rows on each bed with 40-cm spacing. For seed production, plants were harvested at a predefined date post sowing and pods were collected manually. Pods were washed to remove sand residues and shelled. Seeds were sorted and immature seeds with a unique composition (total proteins, oil, and carbohydrates levels of 7, 24, and 63%, respectively) were flash-frozen, and kept at −20°C. For sample standardization, seed samples with the same protein content (2 g) were collected in 50-ml sterile plastic vials. Seeds were lyophilized up to complete drying and kept at +4°C until usage. Upon skin tests, 20 ml of sterile H2O was added to each sample for a 1:10 w/vol final dilution.

### Specific IgE and Specific IgG4

After informed consent, sera from all patients were obtained prior to challenge. Five 5 ml of whole blood in EDTA tubes were drawn, and plasma was extracted for quantification of peanut sIgE and IgE CRD (Ara h 1, 2, 3, 8, and 9) as well as peanut IgG4 and IgG4 CRDs (Ara h1 and Arah2) by ImmunoCAP (Thermo Fisher Scientific Inc., MA, USA).

### Peanut Challenge

All challenges were performed under expert medical supervision. The OFCs to peanut were performed using a commercial preparation of “Bamba” (Osem, Israel), a common snack containing 50% puffed maize and 50% peanut. A portion of 21 Bamba was deemed equivalent to 2.0 g of peanut protein (21). The OFC was performed as an out-patient procedure, starting with a smear of Bamba applied to the buccal mucosa of the lower lip. Subsequently, at 20-min intervals, doses of ¼, ½, 1, 2, 6, and 12 snacks were given. If no reaction occurred during the entire period, the participant was deemed tolerant to peanut (PT). Those who developed acute allergic reactions, such as urticaria, angioedema, vomiting, wheezing, hypoxia, hypotension, and anaphylaxis at any stage of the challenge, were diagnosed as peanut allergic (PA). PA children developing an immediate allergic reaction at a peanut protein dose of <300 mg (1 peanut) were classified as having a low threshold (PALT), whereas those developing allergic symptoms at a dose higher than 300 mg, were classified as high threshold (PAHT).

### Statistical Analysis

Statistical analysis was performed using SPSS 27.0 (IBM SPSS ver 27.0) and JMP Pro 15.0 (SAS jmp pro ver 15.0). For all tests, a *p*-value of <0.05 was considered statistically significant. Continuous variables were described as mean ± 95% confidence interval (CI), and categorical variables as percentages. Comparisons between groups PT vs. PA and PALT vs. PAHT, were analyzed by ANOVA, chi-square test, or Fisher's exact test as appropriate for categorical variables or non-normally distributed continuous variables. Di?erences between the groups were assessed by a putative risk score for OFC outcome. Machine learning techniques were used for a decision tree analysis—this is a non-parametric tool that identifies prediction rules for classifying observations. The algorithm is used to construct classification and regression trees (21, 22), in what is referred to as “supervised learning.” The trees are based on binary splits of covariates at cutoff values that create maximally separated and homogeneous groups. The cutoff of a split is determined statistically. Unlike classical logistic regression, decision tree analysis maximizes the number of data points used. With large data sets, a training set (a subset of the data) is used to construct the tree, and a validation set (the remaining subset) is used to assess and validate its performance. In smaller data sets, repeated random allocations of the data used as the exploratory and validation sets is employed to verify the relative robustness of the decision tree analysis.

## Results

A total of 110 preschoolers with a prior clinical diagnosis of IgE-mediated peanut allergy were enrolled in the study period. There were 73 boys (66%), with an age mean of 40 months (95% CI, 36.6–42.7). After an uneventful peanut challenge, 27 children (24.6%) were labeled peanut tolerant (PT) and advised to reintroduce peanuts into their regular diet. For demographic and clinical data, please see Table 1. On average, parents introduced peanuts into the diet of their children around the age of 10 months (95% CI, 8–12 months). More children in the PA group reported an allergic reaction during their first peanut tasting, 79 vs. 43% in PT children, *p* = 0.043.

**Table 1:** Demographics and clinical presentation of 110 preschool children previously diagnosed with IgE mediated peanut allergy.

	**All** **(*N* = 110)**	**PT** **(*N* = 27)**	**PA** **(*N* = 83)**	***P*-value**
Age (months)	40	35.9	40.9	NS
Gender (%boys)	66%	74%	63%	NS
Atopic dermatitis	64%	71%	61%	NS
Allergic rhinitis	12%	18%	11%	NS
Recurrent wheezing/Asthma	36%	38%	36%	NS
Other food allergies	66%	88%	60%	NS
Age at first exposure to Peanuts (months)	10.3	8.8	10.7	NS
First exposure reaction	74%	43%	79%	0.043
Clinical reaction				
Skin	96%	94%	97%	NS
Respiratory	25%	18%	27%	NS
Gastrointestinal	31%	44%	27%	NS
Anaphylactic	39.4%	37.5	40%	NS

*PT, Peanut Tolerant, i.e., passed a challenge with 2,000 mg of peanut proteins without developing an immediate allergic reaction; PA, Peanut Allergic, i.e., developed an immediate allergic reaction during an observed open challenge with peanut proteins*.

**Figure 1:**
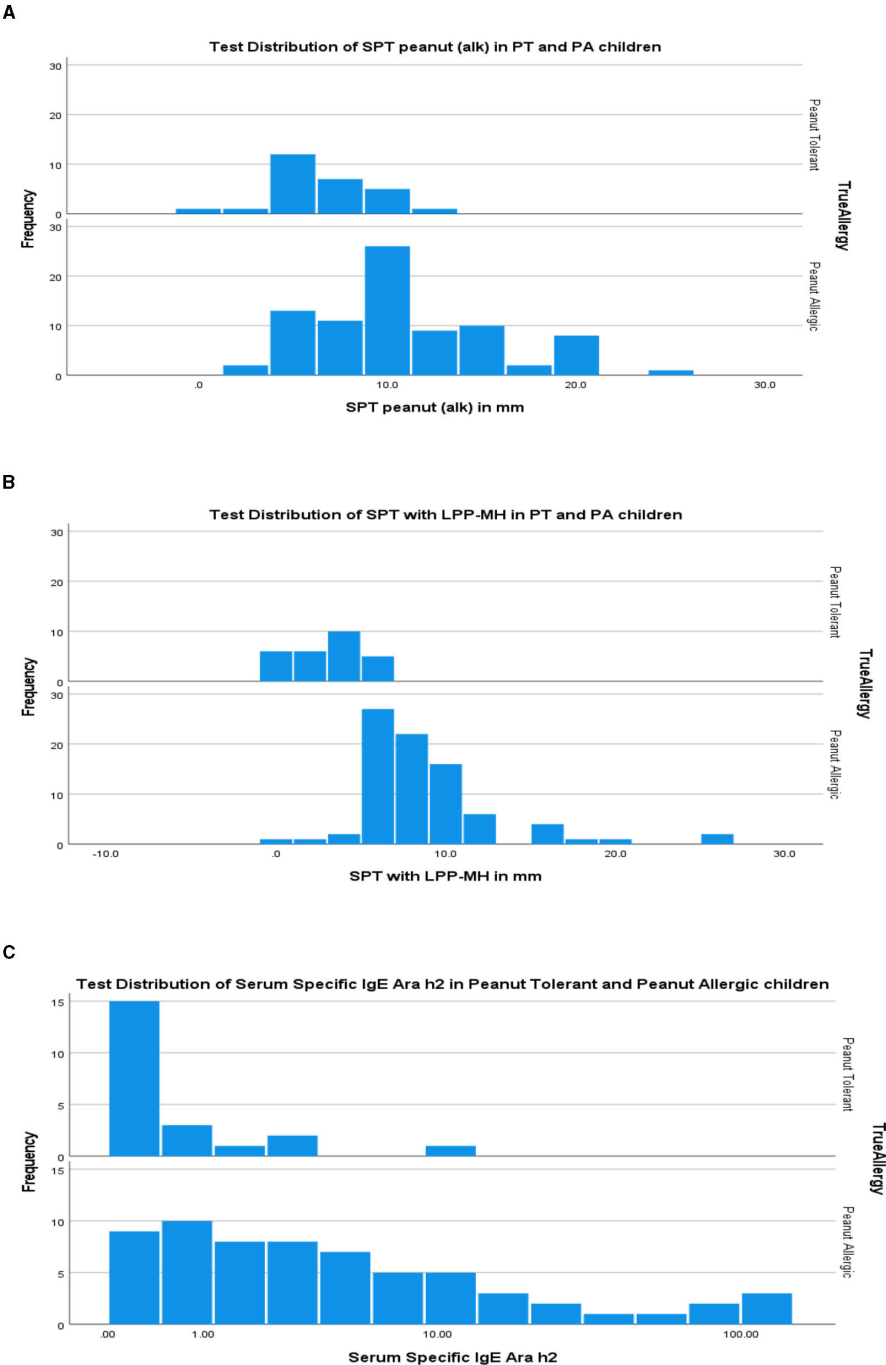
Test distribution of skin tests **(A)** commercial peanut extract (alk), **(B)** LPP-MH and **(C)** Serum Specific IgE to Ara h2 in PT and PA children. PT, Peanut Tolerant, i.e., passed a challenge with 2,000 mg of peanut proteins without developing an immediate allergic reaction; PA, Peanut Allergic, i.e., developed an immediate allergic reaction during an observed open challenge with peanut proteins.

**Figure 2:**
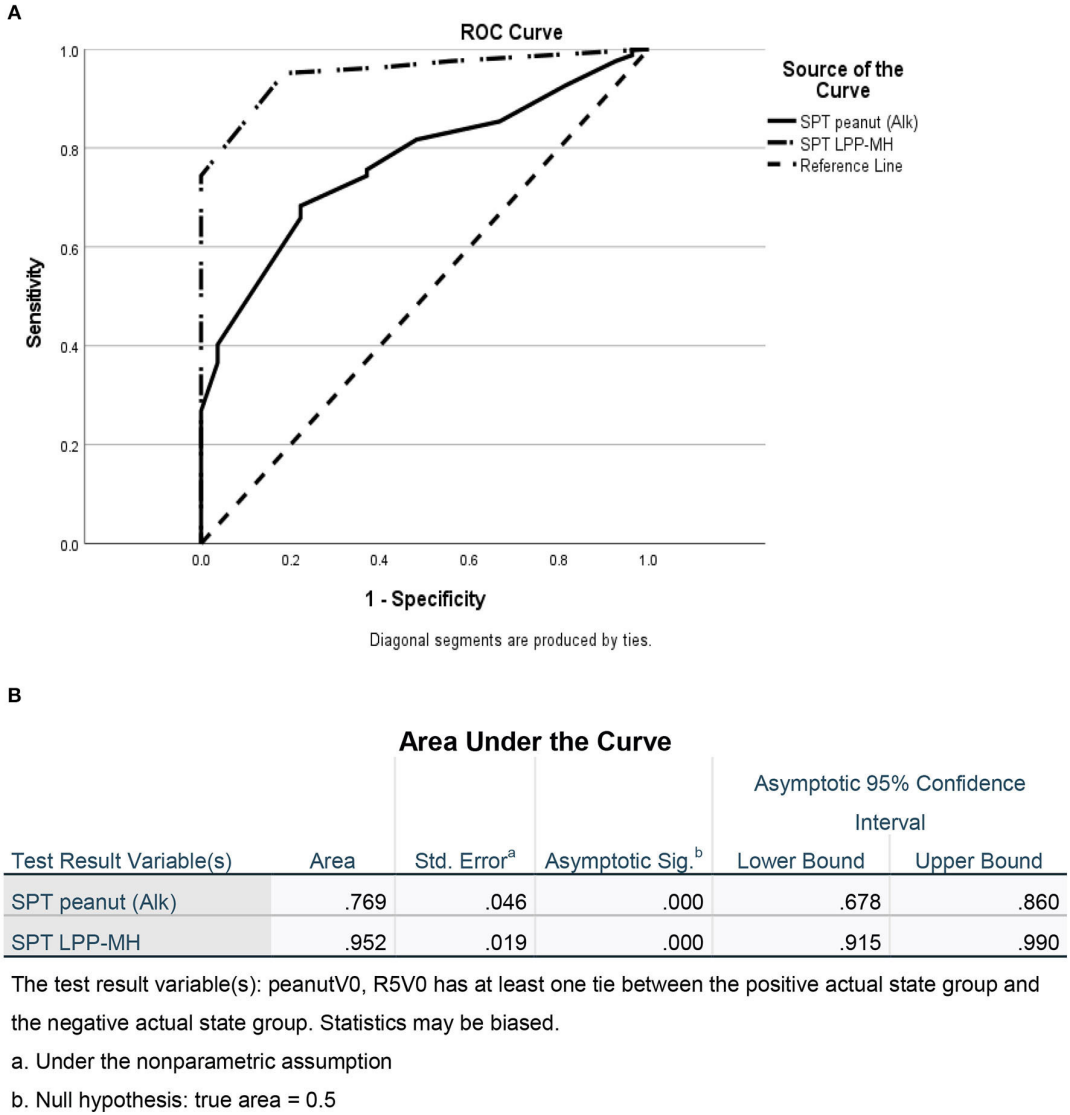
ROC of skin tests with commercial peanut extract and LPP-MH for the diagnosis of Peanut allergy in children. **(A)** ROC curve and **(B)** Area under the curve.

**Figure 3:**
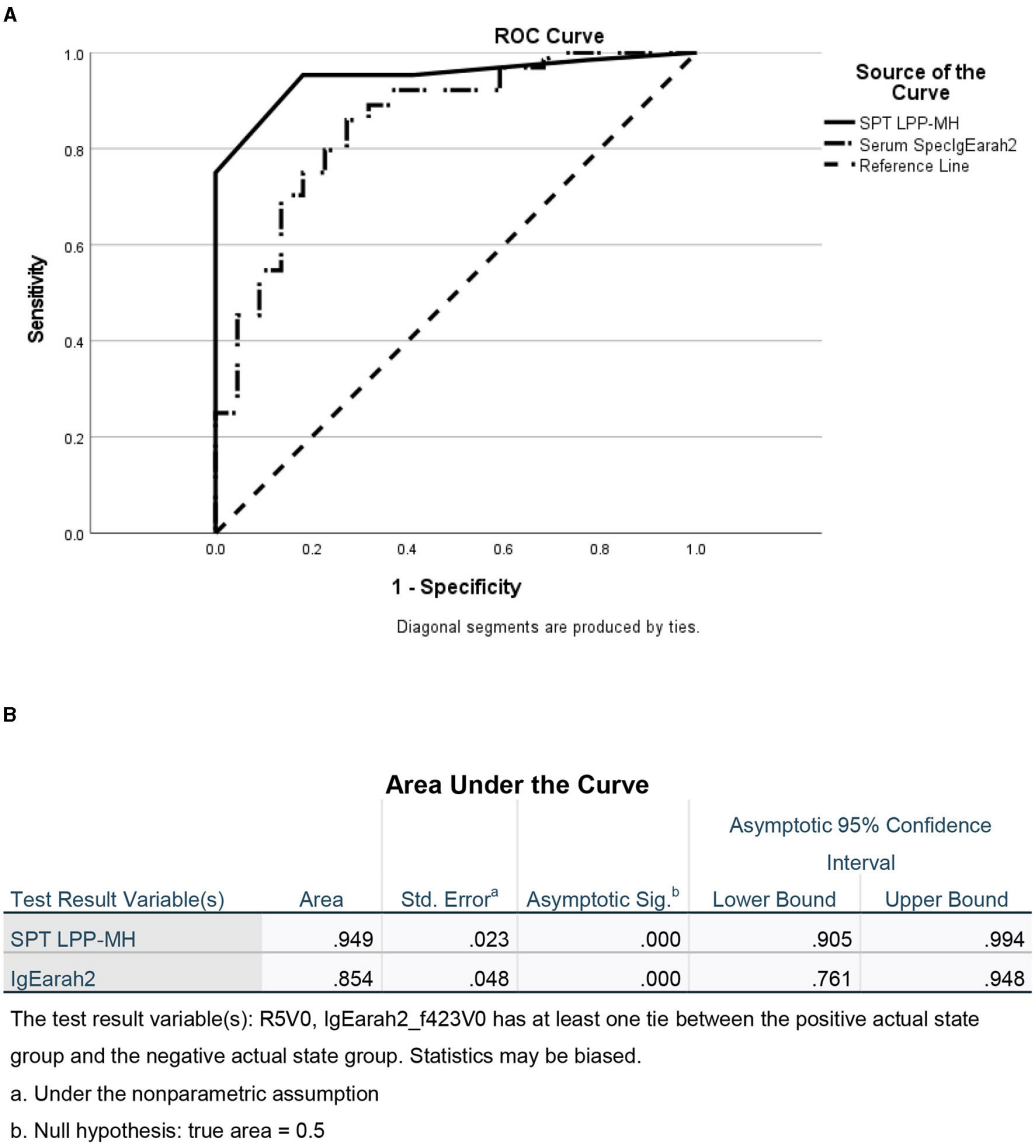
ROC of skin tests with SPT LPP-MH and serum Specific IgE Ara h2 for the diagnosis of Peanut allergy in children. **(A)** ROC curve and **(B)** Area under the curve.

As a group, the classical IgE tests, commercial peanut extracts skin tests, as well as serum-specific IgE to peanuts and peanut components significantly differed between PT and PA children, see Table 2. However, there was also significant overlap between the groups (Figure 1), so that the positive and negative predictive values even at the best cutoff values were only fair (Figures 2, 3). In contrast, the skin prick test with the LPP-MH had better discriminant capabilities as well as better PPV toward the diagnosis of true peanut allergy in this group (see Table 3). The ROC curves comparing peanut SPT vs. LPP-MH SPT and serum-specific IgE to Ara H2 vs. LPP-MH SPT in the diagnosis of peanut allergy are detailed in Figures 2, 3.

**Table 2:** Skin tests, specific IgE and IgG4 to peanuts and peanut allergenic proteins, in 110 preschool children previously diagnosed with IgE mediated peanut allergy.

	**All (*N* = 110)**	**PT (*N* = 27)**	**PA (*N* = 83)**	***P*-value**
**Skin test mm mean (95% CI)**				
Peanut (Alk)	9.8 (8.9–10.7)	6.7 (5.6–7.7)	10.9 (9.8–11.9)	0.0001
LPP-MH (Volcani)	6.9 (6.1–7.8)	2.7 (2.0–3.4)	8.3 (7.4–9.3)	0.0001
**Specific IgE IU/ml mean (95% CI)**				
Peanut	11.8 (6.7–16.9)	2.2 (0.45–3.9)	15.1 (8.4–21.8)	0.03
Ara h1	5.8 (1.6 – 10.0)	0.9 (0.06–1.7)	7.5 (1.9–13.2)	0.17
Ara h2	9.5 (4.9–14.2)	0.92 (0.01–1.9)	12.5 (6.3–18.6)	0.03
**Specific IgG4 ngr/ml mean (95% CI)**				
Peanut	0.71 (0.3–1.1	0.40 (0.01–0.8	0.78 (0.3–1.2)	0.44
Ara h1	0.1 (0.03–0.2)	0.07 (0.01–0.2	0.1 (0.02–0.2)	0.71
Ara h2	0.17 (0.07–0.3)	0.18 (0.01–0.4)	0.17 (0.05–0.3)	0.94

*PT, Peanut Tolerant, i.e., passed a challenge with 2,000 mg of peanut proteins without developing an immediate allergic reaction; PA, Peanut Allergic, i.e., developed an immediate allergic reaction during an observed open challenge with peanut proteins*.

**Table 3:** Sensitivity, specificity and positive and negative predictive values for peanut allergy, of skin tests and serum specific IgE to peanuts or Ara h2, in preschool children with a high suspicion of peanut allergy – i.e., prior probability of PA = 83/110 = 0.75.

**Test (cutoff)**	**Sensitivty**	**Specificity**	**PPV**	**NPV**
**SPT peanut**				
3 mm	0.98	0.09	0.76	0.6
8 mm	0.71	0.82	0.92	0.49
**Peanut IgE**				
0.35	0.92	0.18	0.77	0.43
1	0.83	0.73	0.90	0.59
**Specific Ara h2 IgE**				
0.35	0.89	0.64	0.88	0.66
1	0.73	0.82	0.92	0.5
**SPT LPP-MH**				
3 mm	0.95	0.60	0.88	0.8
5 mm	0.95	0.82	0.94	0.85

**Figure 4:**
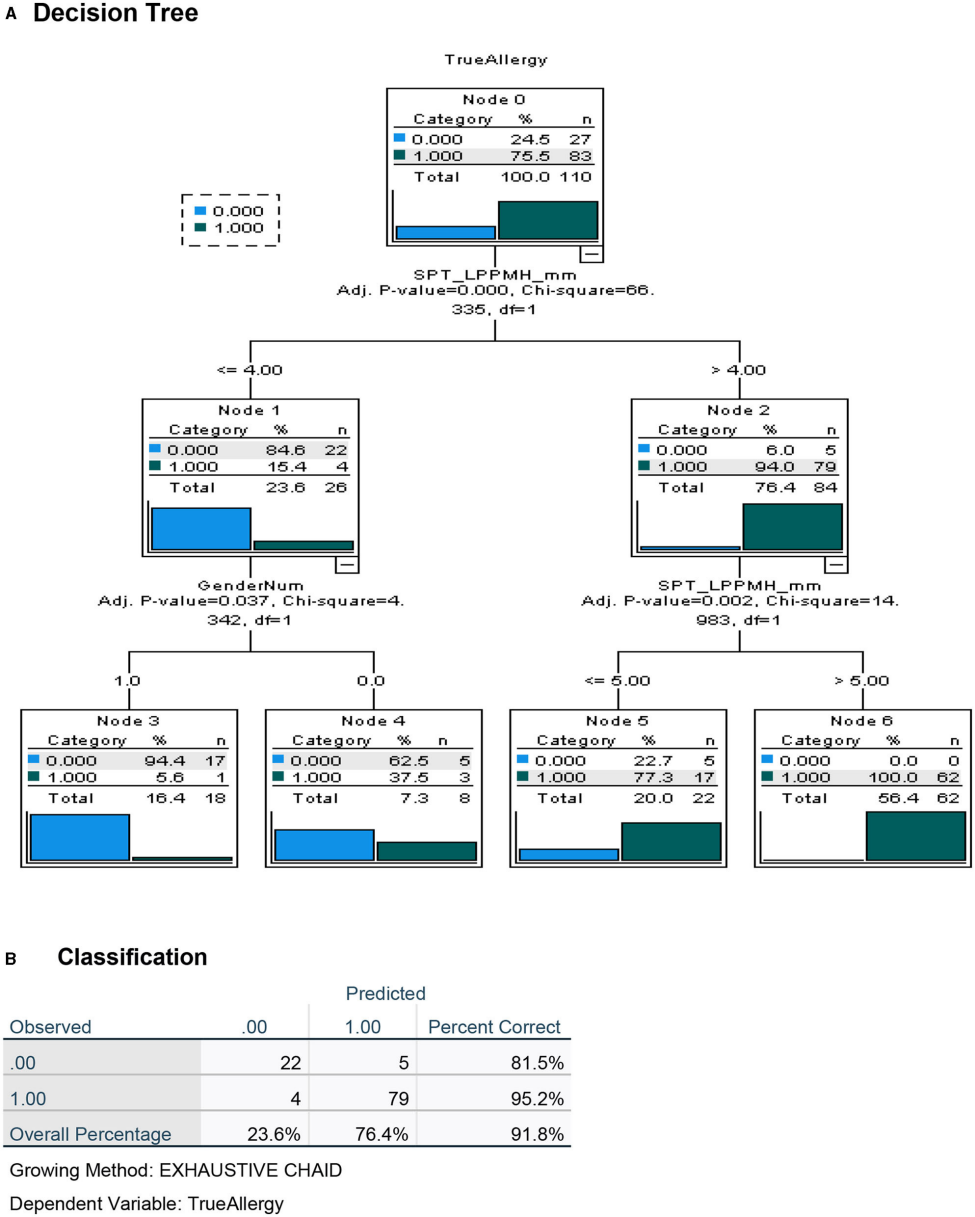
Decision tree analysis for the apriori (before challenge) classification of Peanut Allergy (PA) and Peanut Tolerance (PT) in high risk preschoolers using results of the LPP-MH prick tests. **(A)** Decision tree and **(B)** Classification table.

**Figure 5:**
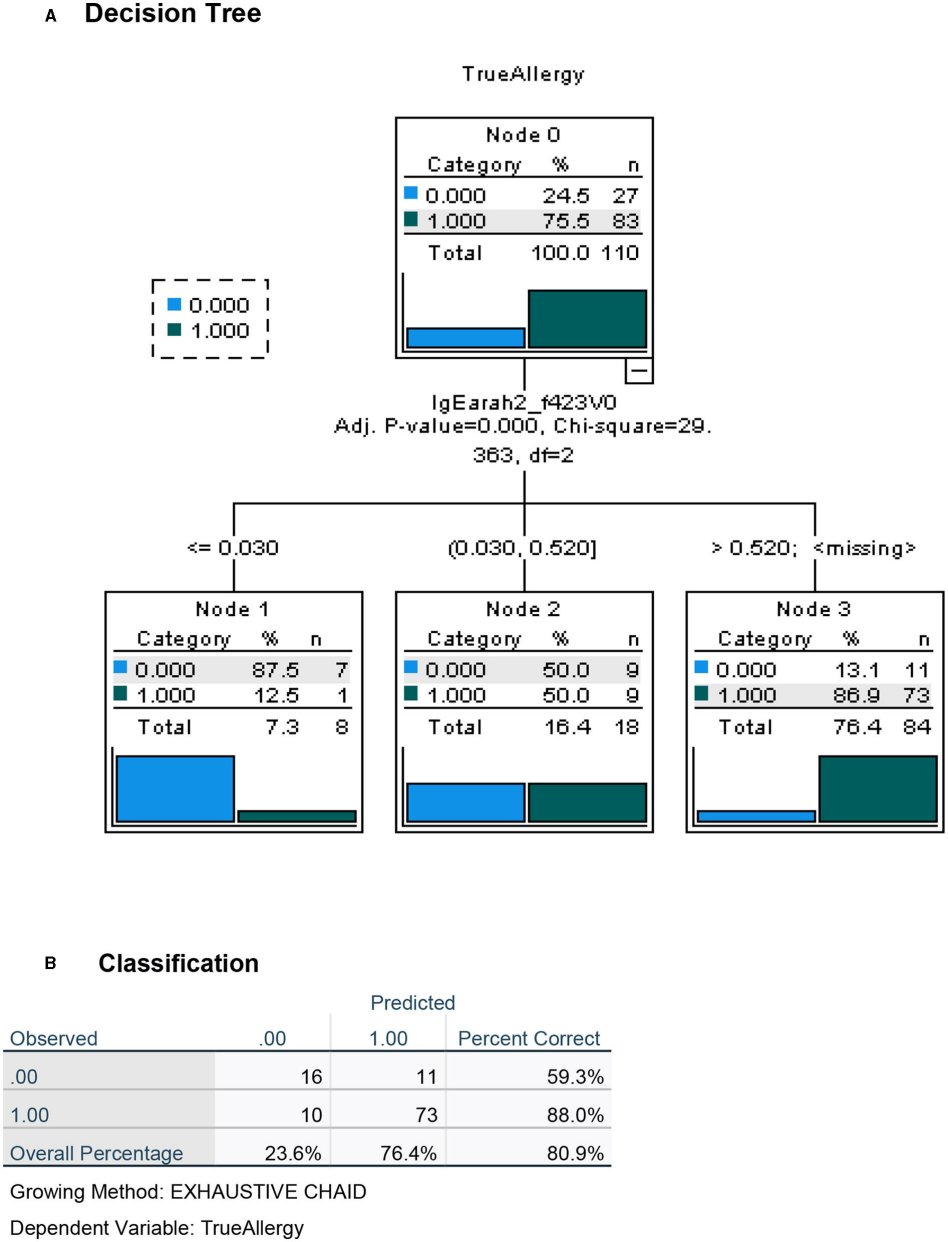
Decision tree analysis for the apriori (before challenge) classification of Peanut Allergy (PA) and Peanut Tolerance (PT) in high risk preschoolers using all other laboratory results but excluding results of the LPP-MH prick tests. **(A)** Decision tree and **(B)** Classification table.

To maximize the discriminatory potential of clinical and laboratory data, and to better predict the outcome of the oral food challenges, we employed a machine learning technique for building a decision tree analysis, a non-parametric tool that identifies prediction rules for classifying observations. The trees are based on binary splits of covariates at cutoff values that create maximally separated and homogeneous groups. The cutoff of a split is determined statistically. Validation of the overall performance of the tree as well as the relative robustness of the decision tree analysis, is achieved by repeated random allocations of the data used as exploratory and validation sets. The decision tree for the diagnosis of PA in this high-risk group of preschoolers was performed both with the inclusion of the test results using the novel LPP-MH prick–prick tests (Figure 4) and with the exclusion of the LPP-MH test (Figure 5). Inclusion of the results of the LPP-MH tests enabled the correct diagnosis of 91.8% of the challenge outcomes using data available in the clinic prior to the performance of a challenge. Simultaneously, if we excluded the data from LPP-MH test, serum levels of Anti Ara h2-specific IgE were the best predictor of the oral challenge outcomes; this however, enabled the correct diagnosis in 80.9% of children.

Peanut allergic preschoolers, reacted on average to a minimal eliciting dose of approximately one-fifth of a gram or <1 peanut kernel (95% CI, 157–287 mg). However, a subgroup of 22 children (27%) exhibited an allergic reaction only after ingesting a dose higher than 300 mg or one whole peanut—this group we labeled as having a “high threshold” PA. In contrast, the remaining group of PA children reacted at an average dose of 78 mg (95% CI, 55–100 mg), or less than a third of a peanut (see Table 4). There were no significant differences between the high and low threshold groups in demographic, clinical, skin-based, or serum-based tests (see Table 4). However, a trend for peanut SPT to be higher in the high threshold group and for Ara h2 IgG4 to be lower in the high threshold group was documented.

**Table 4:** Maximal tolerated doses and minimal eliciting doses for high and low threshold peanut allergic preschoolers.

	**All PA**	**Low threshold**	**High threshold**	***P*-value**
*N* (%)	83	61 (73%)	22 (27%)	—
Maximal tolerated dose in mg (95% CI)	118 (79–156)	37 (25–50)	324 (240–408)	0.0001
Minimal eliciting dose in mg (95% CI)	222 (157–287)	78 (55–101)	600 (475–725)	0.0001
Cumulative dose in mg (95% CI)	391 (271–511)	127 (85–168)	1,085 (847–1324)	0.0001
Age (months)	40.9 (37.5–44.3)	42.3 (38–46.7)	37 (32–42.9)	0.169
Gender (% Boys)	63%	69%	48%	0.08
AD (%)	61%	60%	67%	0.63
AR (%)	11%	12%	8%	0.67
Asthma (%)	36%	38%	29%	0.53
Other food allergy	60%	67%	38%	0.07
**Skin Tests**				
SPT Peanut	10.8 (9.8–11.9)	11.5 (10.2–12.8)	9.2 (7.4–10.9)	0.054
SPT LPP-MH	8.3 (7.4–9.3)	8.6 (7.6–9.7)	7.5 (5.3–9.7)	0.29
**Specific IgE IU/ml mean (95% CI)**				
Peanut	15.1 (8.4–21.8)	17.9 (9.1–26.6)	6.8 (2.2–11.5)	0.16
Ara h2	12.5 (6.3–18.6)	14.8 (6.8–18.6)	5.5 (0.6–10.3)	0.19
**Specific IgG4 ngr/ml mean (95% CI)**				
Peanut	0.78 (0.32–1.24)	0.66 (0.12–1.19)	1.07 (0.07–2.07)	0.42
Ara h2	0.17(0.05–0.28)	0.1 (0.05–0.14	0.33 (0.01–0.73)	0.07

**Figure 6:**
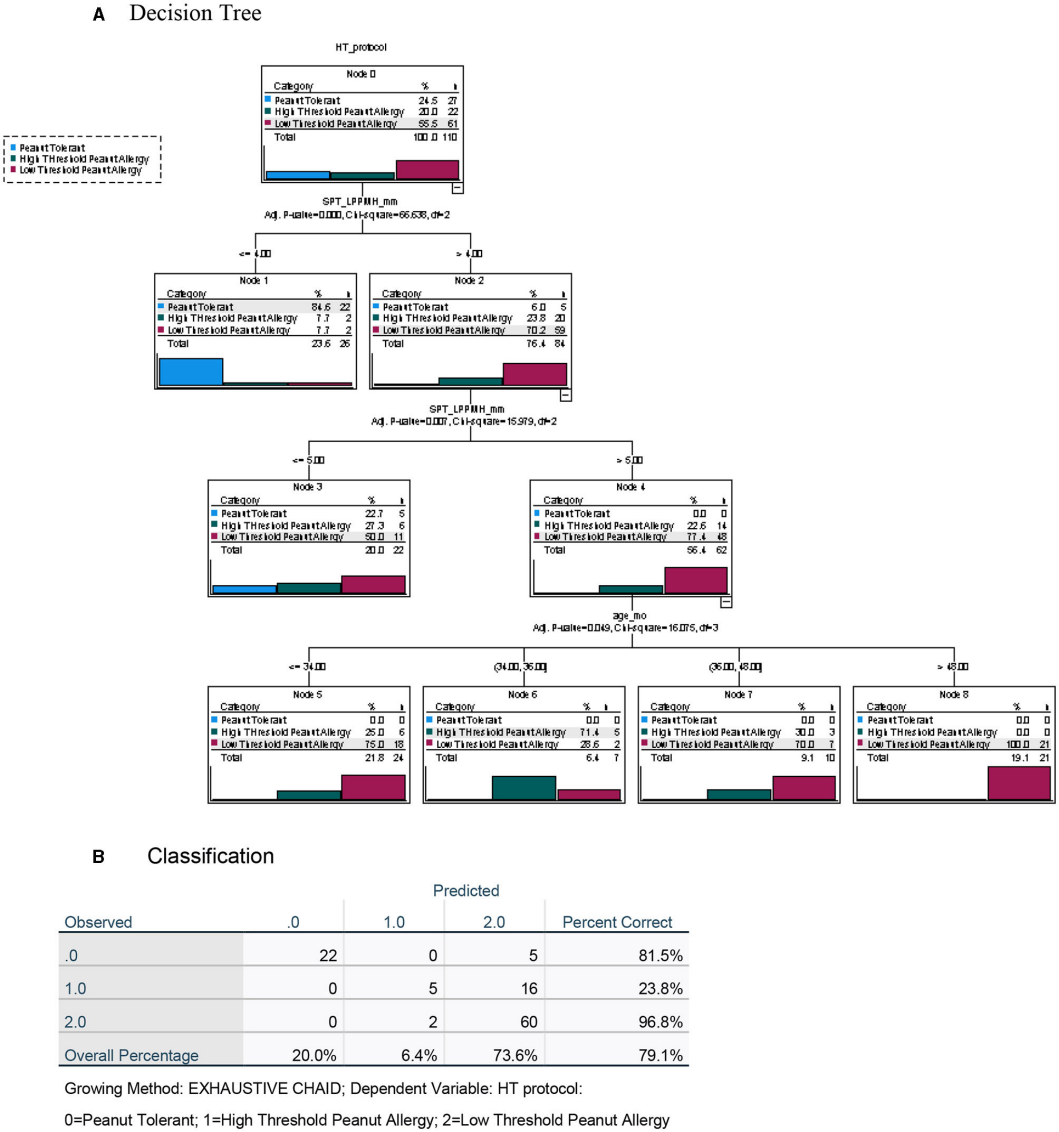
Decision tree analysis for the classification of children with peanut tolerance and peanut allergy, with high (>300 mg) or low (<300 mg) threshold, including the data from the LPP-MH skin tests. **(A)** Decision tree and **(B)** Classification table.

**Figure 7:**
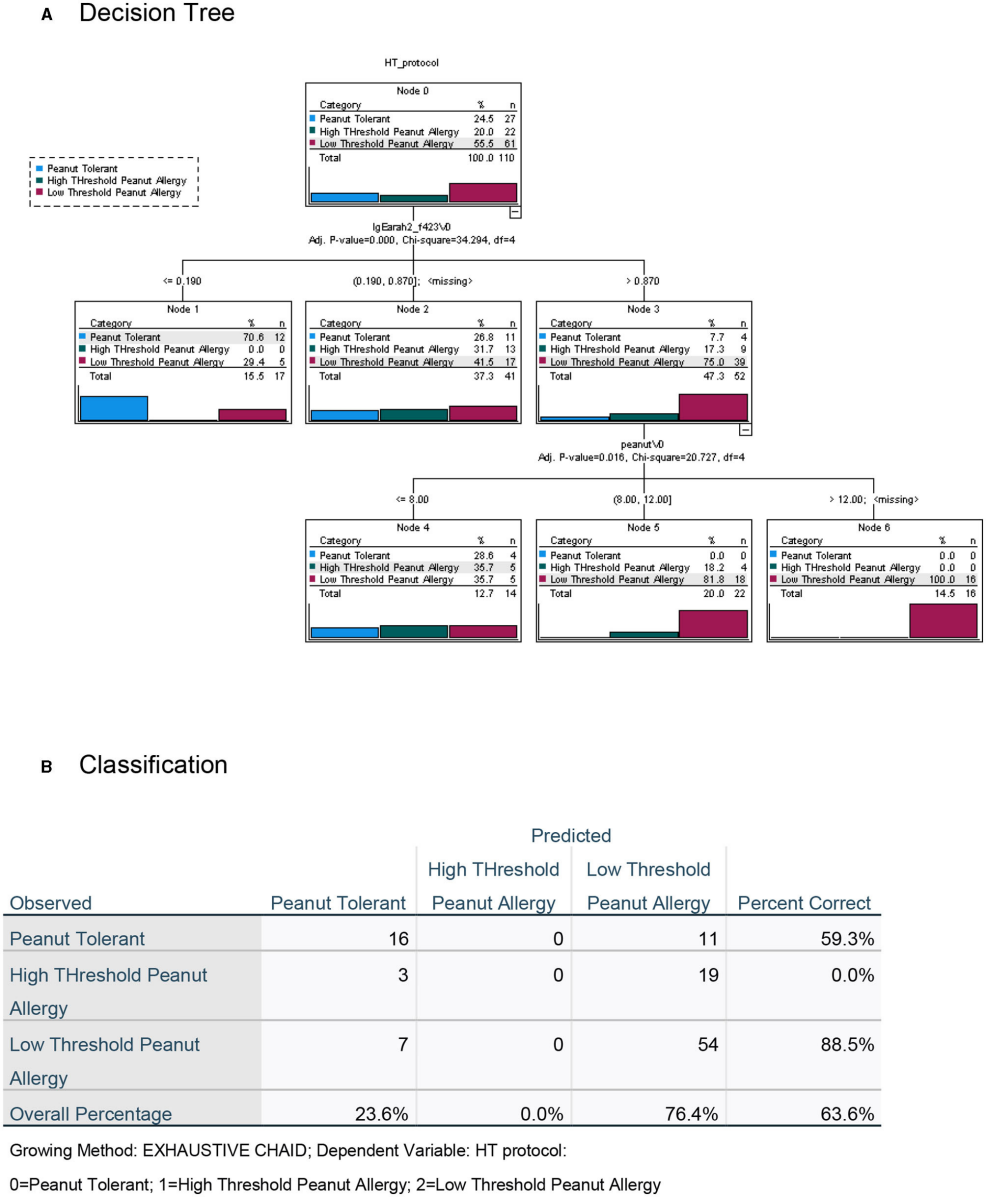
Decision tree analysis for the classification of children with peanut tolerance and peanut allergy, with high (>300 mg) or low (<300 mg) threshold, excluding the data from the LPP-MH skin tests. **(A)** Decision tree and **(B)** Classification table.

To maximize the discriminatory potential of clinical and laboratory data in the diagnosis of high- and low-threshold peanut allergy in this highly allergic group of preschoolers, we employed a decision tree analysis, both with the inclusion of the data from the novel LPP-MH skin tests (Figure 6) and without the LPP-MH results (Figure 7).

Inclusion of the LPP-MH skin test data allowed for the correct classification of more than 97% of the children with severe, low-threshold peanut allergy. The discriminatory algorithm also demonstrates that all of the peanut allergic children older than 4 years of age (22/22) already displayed a severely low minimal eliciting dose, while 14/41 (34%) of those in the younger-aged group belonged to the high threshold group.

Performing the decision tree analysis with the exclusion of the LPP-MH skin tests data centered around the specific Ara h2 IgE, and the results of the skin testing with the commercial peanut extract, however, only allowed for the correct classification of 83.6% of the severely affected, low-threshold peanut allergic children.

## Discussion

An increasing number of young children with suspected allergy to peanuts, presented to the allergy clinic on a regular basis, even in places like Israel where peanuts are not a major food allergen (23). Refuting the diagnosis in a child lacking a relevant history and with negative tests is easily done, with or without a relatively risk-free observed challenge. It is precisely in those young children with a history of immediate allergic reactions and tests demonstrating IgE-mediated sensitization to peanut proteins, deemed correctly as children with a high risk for peanut allergy, where our diagnostic skills are challenged.

While our main focus was avoiding a false-negative diagnosis, i.e., miss-labeling a true positive child and so exposing him or her to another accidental exposure with an allergic reaction, the use of a test with high sensitivity, albeit low specificity, such as the currently used commercial peanut extracts, may have been appropriate. For example, in our group of 110 high-risk preschoolers, a skin test with the commercial peanut extract, at the widely used 3 mm wheal size cutoff, had a sensitivity of 0.98, and a PPV of 76%, in a group with a prior probability of disease of 75% (Table 3). Increasing the positive cutoff level to 8 mm of wheal diameter (optimal value in this study), while decreasing the sensitivity to 0.71, increased the specificity to 0.82 and the PPV to 92%, however rendering the NPV at slightly <50%.

However, a false-positive diagnosis also has severe consequences on the quality of life of patients and families, impacting heightened anxiety from accidental ingestion and limiting participation in social events, school, and work. This falsely incurred diagnosis also increases the risk of developing true life-threatening allergy secondary to long term avoidance (15, 16).

Also, with great strides made toward development of treatment protocols for food allergy in general and peanut allergy in particular (24), we have a new imperative toward safely establishing a minimal eliciting or threshold dose in allergic individuals.

The gold standard for establishing a threshold dose as well as determining the true diagnosis of peanut allergy remains the oral food challenge. Since none of the demographic, clinical, or established office-based tests can reliably predict the outcomes of a food challenge in children at high risk, this type of challenge is deemed risky and requires specialized environments, expensive resources, highly-trained personnel, loss of school and work days, and is rarely performed in young children due to the safety concerns of both parents and physicians.

In this study, we have demonstrated that almost a quarter of preschoolers with a highly suggestive history of immediate allergic reactions after peanut exposure and evidence of skin sensitization to peanut proteins, could safely tolerate 2 g of peanut proteins during an open observed challenge and subsequently, all these children successfully incorporated peanuts into their diets.

The novel prick skin test, LPP-MH, developed by the Volcani Research Institute, exhibited a high sensitivity, specificity, PPV, and NPV of 0.95, 0.82, 94, and 85%, respectively, at a cutoff level of 5-mm wheal size, outperforming the “best established” performance of the serum Ara h2-specific IgE, with a sensitivity, specificity, PPV, and NPV of 0.73, 0.82, 92, and 50%, respectively (see Table 3; Figure 3).

Furthermore, including the results of the skin test with the LPP-MH in an algorithm driven decision tree analysis, we were able to predict correctly, challenge-proven peanut allergy in more than 90% of the children, and severe low threshold peanut allergy in 96.8% of preschoolers at high risk (Figures 6, 7).

The finding that in children with peanut allergy, there may be a window of opportunity below the age of 4 years, where a subset of children, approximately one third in our study, with a relatively high threshold, seems to promote the idea that early identification of this population may enable a safer treatment protocol, a finding supported by previous publications on the safety of such protocols in young children (25, 26).

There are some limitations to our study. The number of children recruited is not overly large. There may be some recruitment bias, as all children were either self-referred or referred by their clinicians for the purpose of enrollment for an interventional study—i.e., treatment of their peanut allergy. Although we routinely recommended a challenge as a diagnostic procedure to all our patience, it may be that extremely severe patients as well as very “mild” patients deemed likely to “outgrow” their allergy were underrepresented on this cohort. A selection bias may not be avoided, as our study was conducted in a tertiary medical center, and so participation was limited to the patients arriving at our doors. Without fail, all preschool children during the study period, with a prior diagnosis of peanut allergy were invited to participate in the diagnostic study, and although not all agreed, only a few declined, so we did not perform statistical comparisons. All costs were covered by the study grant so we did eliminate a financial bias in participation.

The challenge procedure was open as opposed to blinded and did not use minutely weighted peanut flower with a known protein content. The open OFC were performed using a commercial preparation of “Bamba,” starting with a smear of the snack applied to the buccal mucosa of the lower lip, and then, at 20-min intervals, doses of ¼, ½, 1, 2, 4, 6, and 12, approximately equivalent to 1, 20, 40, 80, 160, 320, 480, and 1,000 mg. The idea of such a “real life” challenge is that of having a child react to one of the first three doses, i.e., a minimal eliciting dose of less than one-tenth of a peanut, regardless of the “true” eliciting dose, increases the risk of the patient to accidental exposures and reactions even to “may contain” labeled foods. It is also true that we had a number of challenges with what is called “left censored” results, i.e., patients reacting to the first lip dose estimated as 1 mg of peanut protein. Therefore, our estimates of the minimal eliciting dose in the severe group of low threshold sensitivity with a calculated mean 78 mg (95% CI 55–101) may in fact be a slightly optimistic evaluation.

Also, the study was performed in one center with a relatively homogenic population, therefore for the validation of the skin prick test with LPP-MH for the pre-challenge diagnosis of both peanut allergy and low threshold peanut allergy in children, additional multicenter, studies are needed.

As well, the number of children within the high threshold level peanut allergy is too small to enable robust predictive conclusions, so that the decision tree analysis, although including an internal statistical validation, requires validation in larger scale studies.

In addition, the question of whether data from one study can be reproducible in other environments and what if any of the published knowledge can be generalized as a universal take home message, is a subject to intense debate in the scientific community (27). To mitigate this, we have presented a series of statements, as a formal application of Meaning Equivalence Reusable Learning Objects (MERLO) statements to define a boundary of meaning (BOM) representing a generalizability of findings in this paper. The BOM represents what can and what cannot be inferred from the study and enables researchers to design studies that are capable of challenging or reproducing the published results (Table 5).

**Table 5:** Generalization of study findings.

**Target statement**	**Meaning Equivalence (MEF)included in BOM**	**Surface Similarity (SSF)not included in BOM**
Finding 1: A subgroup of preschool children with a history of allergic reactions and sensitization to peanuts, have outgrown their peanut allergy and can successfully reintroduce peanuts in their diet.	Preschool children can outgrow their peanut allergy even while maintaining sensitization to peanut proteins	All children with a diagnosis of peanut allergy, should immediately undergo a diagnostic challenge.
	In a group of preschoolers with a prior history of allergic reaction to peanuts, positive skin tests to commercial peanut preparations are NOT a guarantee for continuing peanut allergy	Skin tests with commercial peanut extracts should not be performed.
	Reintroduction of peanut proteins into the diet, after an observed challenge, is possible in a subgroup of high risk preschoolers with a history of allergic reaction to peanuts.	High levels of positive skin tests or IgE sensitization to peanuts or peanut components, are an absolute contraindication to performing a peanut challenge
Finding 2: A simple skin test with a novel lyophilized peanut preparation, LPP-MH, offers better sensitivity, specificity, PPV and NPV for peanut challenge outcomes, ie: diagnosis of Peanut Allergy	Skin tests are the bedrock of allergy practice and diagnosis	Skin tests with commercial peanut extracts should not be performed
	There is a need for improving the sensitivity, specificity and predictive values of “bedside” tests used in the diagnosis of food allergy	The sensitivity, specificity and predictive values of allergy tests are intrinsic values. Therefore, there is no need to reassess for my own patients in my own clinic.
	A novel prick skin test with LPP-MH enables a pre-challenge diagnosis of peanut allergy in high risk children, potentially reducing the need for diagnostic high risk challenges	An improved bedside diagnostic test, with high negative predictive value, can enable performance of peanut challenges in clinic, even in the absence of appropriate facilities and trained personnel.
Finding 3: A subgroup of peanut allergic toddlers display a high threshold of sensitivity, however this high threshold profile seems to disappear after 4 years of age.	In a subgroup of peanut allergic toddlers, the minimal eliciting dose may be higher than 1 peanut.	Peanut allergic toddlers (3 years of age or younger), do not have severe peanut allergy
	In the subgroup of peanut allergic patients with a high threshold, Oral Immunotherapy with subthreshold doses may be a safe and feasible solution	If the parents or caregiver of peanut allergic children are not interested in a desensitization treatment, there is no need for a peanut challenge.
	Peanut allergic children older than 4 years of age, seem to display a very low eliciting dose, usually <1/5 of a peanut.	

*We present a formal application of Meaning Equivalence Reusable Learning Objects (MERLO) statements to define a boundary of meaning (BOM) representing a generalizability of findings in this paper. The BOM represents what can and what cannot be inferred from the study and enables researchers to design studies that are capable of challenging or reproducing the published results*.

## Conclusions

Almost one-quarter of preschool children, with a history of allergic reactions to peanuts and positive standard IgE-mediated tests for peanut allergy, can tolerate the reintroduction of peanut proteins into their diet after resolving their allergy and, thus, can avoid adverse health outcomes associated with the false diagnosis.

Also in the younger age group, a quarter of the peanut allergic children, display a relatively high threshold, potentially enabling an easier and safer oral immunotherapy protocol in this window of opportunity in childhood.

The use of the novel diagnostic skin test, LPP-MH, significantly improves the predictive value of outpatient evaluation for the outcomes of peanut challenge as well as the expected threshold at which the PA child will react, thus, making for a better-informed decision of how, when, and where to challenge.

## Data Availability Statement

The raw data supporting the conclusions of this article will be made available by the authors, without undue reservation.

## Ethics Statement

The studies involving human participants were reviewed and approved by Sheba Medical Center ERB, Tel Hashomer, Israel. Written informed consent to participate in this study was provided by the participants' legal guardian/next of kin.

## Author Contributions

MK and RH were responsible for the inception and implementation of the study, RK to the statistical design and analysis, and all clinicians for the supervision of children during testing and challenge procedures. All authors contributed to the clinical or scientific content.

## Funding

All the research was performed using publicly awarded funding from the Chief Scientists Office, Ministry of the Economy and Ministry of Agriculture.

## Conflict of Interest

The authors declare that the research was conducted in the absence of any commercial or financial relationships that could be construed as a potential conflict of interest.

## Publisher's Note

All claims expressed in this article are solely those of the authors and do not necessarily represent those of their affiliated organizations, or those of the publisher, the editors and the reviewers. Any product that may be evaluated in this article, or claim that may be made by its manufacturer, is not guaranteed or endorsed by the publisher.
